# Ultrasound-Guided Percutaneous Lavage for the Treatment of Calcific Tendinopathy of the Supraspinatus: A Comprehensive Approach

**DOI:** 10.7759/cureus.79515

**Published:** 2025-02-23

**Authors:** Carolina P Carvalho, Mariana G Xavier, André R Pereira, Sofia G Silva, André O Cruz

**Affiliations:** 1 Physical Medicine and Rehabilitation, Unidade Local de Saúde do Alto Minho, Viana do Castelo, PRT

**Keywords:** calcific tendinitis of shoulder, percutaneous needle aspiration and lavage, shoulder pain, supraspinatus tendon, ultrasound-guided

## Abstract

Calcific tendinopathy of the supraspinatus is a common shoulder condition, characterized by calcium deposits in the supraspinatus tendon, part of the rotator cuff. This condition leads to pain, functional limitations, and a reduced quality of life. Its etiology is multifactorial, involving degenerative processes, metabolic alterations, and biomechanical factors. Ultrasound-guided percutaneous lavage (UGPL) is an emerging treatment option to remove calcium deposits and restore function. The shoulder joint's anatomy is intricate, and calcific tendinopathy primarily affects the supraspinatus tendon, causing impingement and pain. Pathophysiologically, calcific tendinopathy is thought to involve fibrocartilaginous metaplasia, where tendon cells transform into cartilage-like cells, facilitating calcium deposition. The condition progresses through three stages: pre-calcific, calcific, and post-calcific, with varying symptoms and responses to treatment. Epidemiologically, calcific tendinopathy of the rotator cuff is more prevalent in individuals aged 30-50 and is often linked with other medical conditions, such as diabetes and cardiovascular diseases. Diagnosis is typically confirmed through imaging, including radiographs, ultrasound, and magnetic resonance imaging. While conservative treatments such as non-steroidal anti-inflammatory drugs, physical therapy, and shockwave therapy are effective for many patients, some cases require more invasive interventions. UGPL is a minimally invasive procedure that has demonstrated good short- and medium-term outcomes. This technique, which involves needle insertion into the calcified deposits under ultrasound guidance to fragment and aspirate the calcium, offers significant symptomatic relief and restores shoulder function. However, challenges remain, particularly with dense or small calcifications, and the long-term effectiveness of the procedure is still uncertain. Calcific tendinopathy of the rotator cuff requires a personalized treatment approach. UGPL has shown promise as an effective and safe option for patients who do not respond to conservative treatments, such as rehabilitation programs. However, further research is needed to refine treatment protocols, improve patient selection, and assess the long-term efficacy of the procedure. A multidisciplinary approach is essential to optimize patient outcomes and manage this challenging condition effectively.

## Introduction

Calcific tendinopathy of the rotator cuff (CTRC) is a complex condition whose pathogenesis is not fully understood. The supraspinatus tendon is the most commonly affected, leading to subacromial impingement and mechanical limitation at the shoulder level and beyond. Several theories have been proposed to explain the underlying processes, highlighting the role of fibrocartilaginous metaplasia and cellular calcification [1-3]. The progression of CTRC is divided into three stages: (1) pre-calcific phase; (2) calcific phase; and (3) post-calcific phase [2,3]. These stages reflect a dynamic process that can range from an asymptomatic phase to severe pain, with spontaneous resolution in some cases [2,3].

Some studies have reported prevalence rates ranging from 2.7% to 22%, primarily affecting individuals between the ages of 30 and 50. No deposits were found in elderly individuals [4]. Although CTRC is often an asymptomatic finding in imaging studies, in patients with shoulder pain, it is identified in 6.8% of cases [5]. The incidence varies between 2.7% and 20%, as reported by different authors. In approximately 10-20% of patients, deposits are bilateral. Most studies have found a higher incidence in women compared to men [4]. Researchers also agree that deposits are most frequently localized in the supraspinatus tendon [4,5].

The main clinical feature of CTRC is shoulder pain, which can be either acute or chronic. This pain may occur with or without gradual or sudden restriction of joint mobility. Acute symptoms are often attributed to various causes. According to Uhthoff et al. [6], pain is frequently associated with the resorption phase, probably due to an inflammatory response that facilitates the removal of calcium deposits. In 1941, Bosworth reported that symptoms were more common when the calcifications exceeded 1.5 cm in diameter, a finding confirmed by later studies [7].

The physical examination of the shoulder includes medical history, inspection, palpation, range of motion (ROM) assessment, and muscle testing [3]. Radiological investigations (e.g., conventional radiographs in anteroposterior, lateral, and outlet views) confirm the diagnosis and, in some cases, identify it even in asymptomatic patients. They also allow for determining the phase of the condition and monitoring its progression [8]. Ultrasound examination has proven to be more sensitive in identifying calcium deposits in the rotator cuff. On the other hand, computed tomography allows for better localization of the deposits. Although conventional radiographs can detect the deposits, magnetic resonance imaging (MRI) provides a more detailed evaluation of coexisting pathologies [8]. Bianchi et al. classified calcifications ecographically into three different types based on their morphology and echographic structure: type I calcification - hyperechoic focus with a well-defined posterior acoustic shadow; type II calcification - the posterior acoustic shadow is faint; and type III calcification - it appears as a hyperechoic focus without a posterior acoustic shadow, poorly defined, with echoes within it [9].

Non-surgical treatment is the first option in managing calcific tendinitis of the shoulder. It includes the use of non-steroidal anti-inflammatory drugs (NSAIDs), physiotherapy, ultrasound-guided aspiration, and extracorporeal shock wave therapy (ESWT). The failure of non-surgical treatment is defined as the persistence of symptoms after at least six months of conservative rehabilitation treatment, including three months of standardized therapy [4,7]. In the acute phase, NSAIDs are recommended to relieve pain, and physiotherapy (passive ROM exercises) is advised to prevent shoulder stiffness. The use of local steroid injections at this stage is controversial, with studies showing positive, neutral, or even negative effects, as they may halt the resorption of deposits. In most cases, conservative treatment is sufficient to resolve the symptoms [5,7].

Ultrasound-guided percutaneous lavage (UGPL) is a minimally invasive technique that is increasingly used to treat CTRC. Farin et al. [10] were the first to describe the results of this procedure, reporting 73% excellent results associated with a reduction in the size of the calcifications [7]. Most studies have reported good short- and medium-term results with this technique. Despite the promising results, further long-term studies with larger populations and better-defined protocols are needed. Due to variability in methods and the low quality of evidence, especially due to the lack of control groups in many studies, the efficacy of this technique cannot be confirmed with certainty [7].

The aim of this technical report is to analyze the clinical, diagnostic, and therapeutic aspects of calcific tendinopathy of the supraspinatus, with a special focus on the efficacy and role of the lavage technique as a therapeutic tool.

## Technical report

Proposed UGPL (according to the procedures practiced in our local health unit (Unidade Local de Saúde do Alto Minho, Portugal)

**Figure 1:**
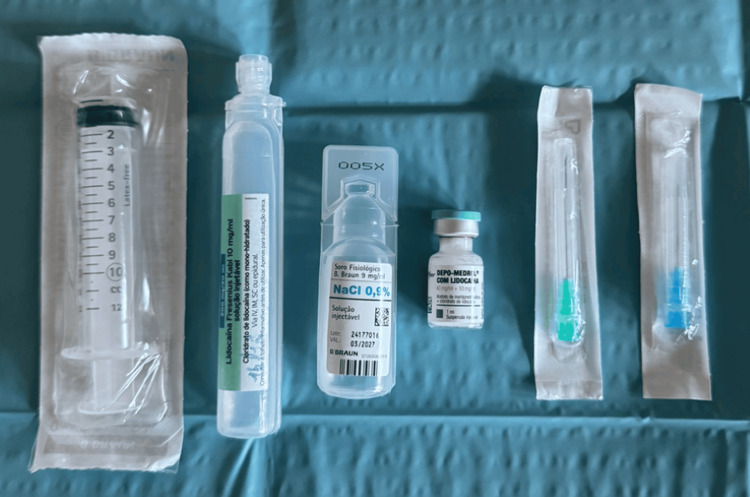
Necessary materials for performing UGPL UGPL: Ultrasound-guided percutaneous lavage This is an original image.

The materials required to perform the procedure are sterile gloves, antiseptic skin solution sterile ultrasound gel, an ultrasound machine with a 7-13 MHz linear probe, syringes (preferably luer-lock) of 5 or 10 mL, a local anesthetic (lidocaine at 1-2%; saline solution), 23 or 25 G needle for local anesthesia, 18 or 20 G needle for irrigation, corticosteroid, gauze, and dressing (Figure 1).

Description of the procedure

**Figure 2:**
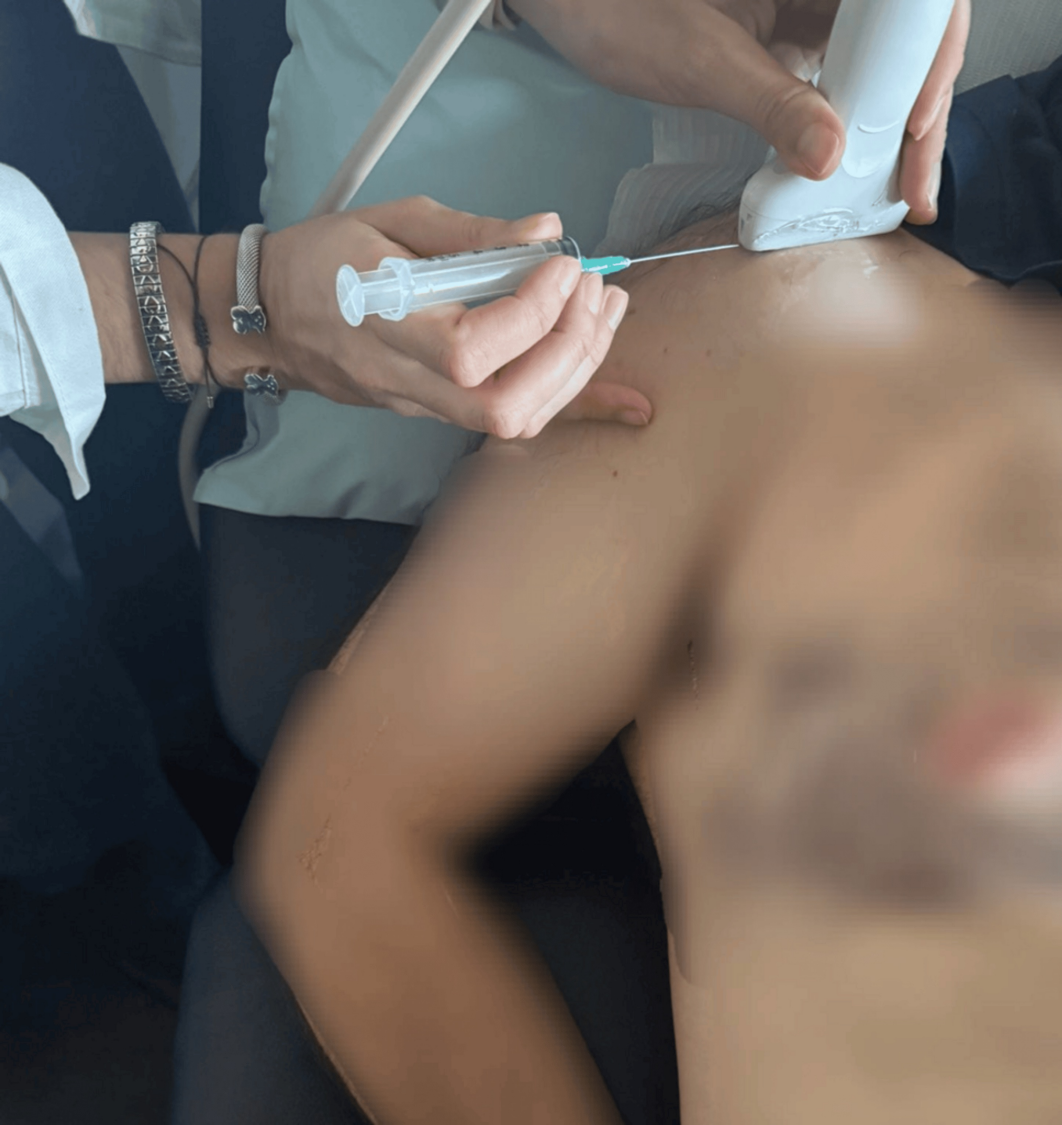
Exemplification of patient positioning for performing the procedure This is an original image.

The patient should be placed in a supine position or with a slight incline, ensuring the arm is in either internal or external rotation, depending on the location of the calcification. The arm should also be slightly abducted to provide optimal access to the affected area (Figure 2). It is recommended to first locate the center of the calcification in the short-axis view of the transducer. Once the calcification is identified, the probe should be rotated to the long axis to allow for proper visualization of the needle's trajectory and ensure accurate placement. The procedure can be painful, although it depends on each individual's perception of pain.

Procedure

We begin the treatment with intramuscular or subcutaneous administration along the planned path to reach the calcification, using 4 cc of 2% lidocaine. In this case, it was not necessary to repeat the local anesthesia, but it can be done during the procedure, if necessary.

**Figure 3:**
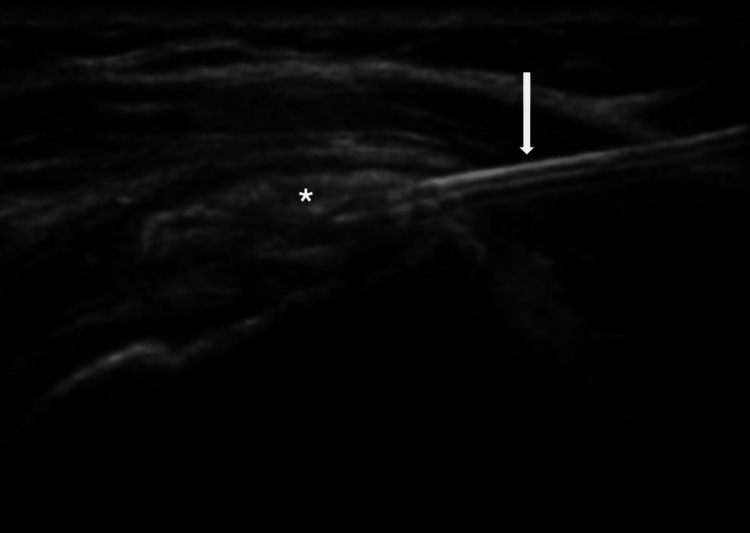
Ultrasound image of the needle (arrow) entering the calcification (asterisk) This is an original image.

**Figure 4:**
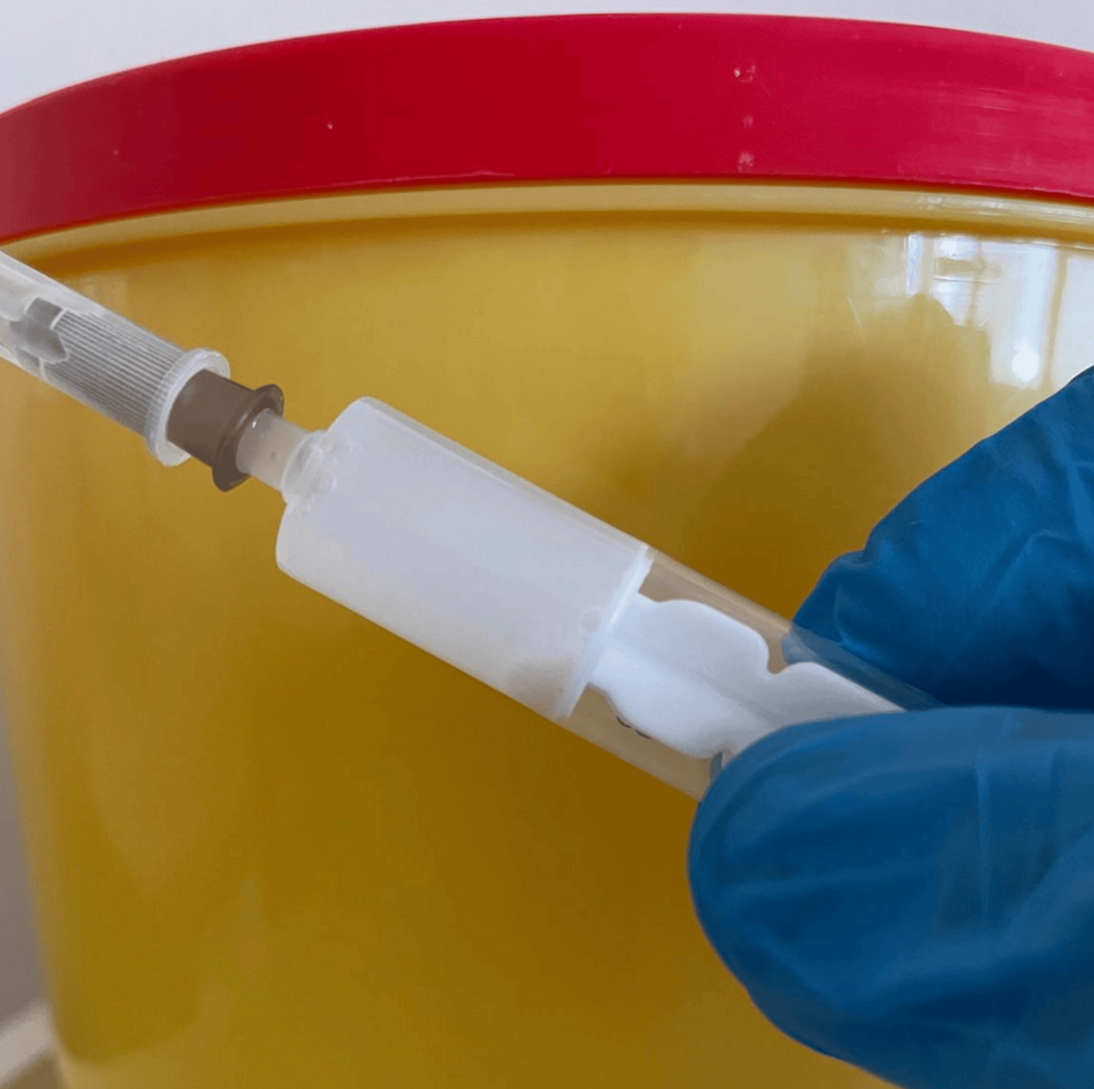
Syringe with washed calcium from a patient This is an original image.

Next, the calcification is traversed using a needle large enough to allow the release of the calcified material, guided by ultrasound in the longitudinal axis (Figure 3). Once the needle tip is correctly positioned, small amounts of a mixture of saline solution and lidocaine are injected into the calcification. This facilitates its rupture, allowing the calcified material to flow through the needle (Figure 4). This process is achieved by releasing pressure on the plunger, allowing the cloudy liquid, a mixture of saline and calcium, to move back into the syringe.

When the aspirated liquid becomes cloudy, the syringe is replaced with another one filled with saline solution, ensuring the needle stays in its original position. This procedure can be repeated two or three times if necessary. It is crucial to keep the needle in place throughout the entire process.

In some cases, particularly if the calcification is small or very dense, it may not be possible to extract the calcium. In such situations, perforations may be made within the calcification to fragment it, facilitating its subsequent natural reabsorption.

At the end of the procedure, the needle is removed, and 1 or 2 mL of depot corticosteroid (e.g., triamcinolone) is injected into the subacromial bursa. This is done because any calcified material that may have escaped into the bursa could lead to painful bursitis. The time required to do all the procedures depends on each professional's experience, but it is expected to take a few minutes (in our case, it took 15 minutes).

Post-procedure recommendations

Once the treatment is completed, immobilization of the limb is not recommended, but it is advised to avoid intense physical activities for a period of one to two weeks. It is important to inform the patient about the possibility of experiencing pain once the anesthetic effect wears off. This pain may be more intense if a significant amount of calcium has accumulated in the bursa, so analgesic treatment should be prescribed for the following days.

In most cases, significant improvement is observed during the first four weeks after the procedure. The calcification typically decreases considerably, especially when it has been successfully ruptured. However, it may take longer in cases where the calcification has not been fully aspirated, and it will depend on the natural reabsorption of the calcification.

A clinical evaluation will be performed after two weeks to determine whether a repeat treatment is necessary, particularly if symptoms persist or if calcified material that can still be extracted is found.

The results are usually favorable, with a reduction in pain and greater reabsorption of the calcification. In this case, the patient reported a reduction in pain following the procedure, with the numerical pain scale score decreasing from 7/10 to 4/10.

Regarding complications, they are rare but may include mild vagal episodes during the washing process. Minor complications such as bursitis and temporary restrictions in joint mobility have also been reported.

## Discussion

CTRC remains a significant clinical challenge due to its multifactorial etiology and the varying degrees of severity experienced by patients. This condition is primarily characterized by the accumulation of calcium deposits within the supraspinatus tendon, a critical component of the rotator cuff, leading to pain, functional disability, and reduced quality of life. Although the exact pathophysiological mechanisms remain elusive, current theories emphasize the importance of degenerative processes, fibrocartilaginous metaplasia, and altered metabolic states in the formation and resorption of calcifications [2,3]. This complexity calls for a comprehensive and personalized approach to diagnosis and treatment, tailored to the individual’s clinical presentation.

The epidemiology of CTRC highlights its higher prevalence in individuals aged 30-50, with women being more commonly affected than men [4]. Additionally, comorbidities such as diabetes, thyroid disorders, and cardiovascular diseases are frequently associated with the condition, suggesting a possible systemic influence [5]. Notably, while many cases of calcific tendinopathy are asymptomatic, others present with severe symptoms that can significantly impair shoulder function. This spectrum of presentation underscores the necessity for a nuanced approach to treatment [7].

Conservative management remains the first-line treatment, with NSAIDs and physical therapy often providing satisfactory relief for most patients [4,7]. However, conservative measures may fail, particularly in cases with larger deposits, bilateral involvement, or deposits in the anterior portion of the acromion. When these measures prove ineffective, interventional procedures, such as UGPL offer a promising alternative, with studies showing good short- and medium-term outcomes [4]. As a minimally invasive technique, UGPL allows for precise targeting and removal of calcium deposits, providing symptomatic relief and restoring shoulder functionality [10].

The procedure involves the insertion of one or two needles into the calcific deposits under real-time ultrasound guidance. The technique has shown promising results, particularly when guided by the two-needle method, where fluid is injected to fragment the calcification and aspirate it into a syringe [7,11]. Corticosteroid injections at the end of the procedure help mitigate the risk of postoperative bursitis, a common complication. Post-procedure, most patients experience significant improvement in pain and mobility, with further clinical evaluations ensuring adequate calcium reabsorption [7,11].

Despite its efficacy, the UGPL technique is not without its limitations. One of the most significant challenges is the variability in patient responses, particularly with small or very dense calcifications that may resist aspiration. Additionally, the long-term benefits of UGPL remain uncertain, as follow-up studies suggest that the benefits of this treatment may diminish over time. Further studies with larger sample sizes and extended follow-up periods are essential to evaluate the long-term efficacy and potential complications of this technique [7].

## Conclusions

The treatment of calcific tendinopathy of the rotator cuff requires a tailored approach, combining non-surgical therapies with interventional techniques such as UGPL when necessary. The success of UGPL as a treatment option highlights its role as a safe and effective procedure for patients with refractory symptoms. However, the need for further research is evident, particularly in establishing standardized protocols, refining the indications for its use, and understanding the long-term outcomes. A multidisciplinary approach involving clinicians, physiotherapists, and radiologists is essential for optimizing patient outcomes and improving the management of this debilitating condition.
